# Chronic, Elevated Maternal Corticosterone During Pregnancy in the Mouse Increases Allergic Airway Inflammation in Offspring

**DOI:** 10.3389/fimmu.2019.03134

**Published:** 2020-01-21

**Authors:** Arianna L. Smith, Emmanuel Paul, Devin McGee, Ranuka Sinniah, Emily Flom, Devan Jackson-Humbles, Jack Harkema, Karen E. Racicot

**Affiliations:** ^1^Department of Obstetrics, Gynecology and Reproductive Biology, College of Human Medicine, Michigan State University, Grand Rapids, MI, United States; ^2^Department of Pathobiology and Diagnostic Investigation, College of Veterinary Medicine, Michigan State University, East Lansing, MI, United States

**Keywords:** stress, pregnancy, prenatal programming, allergic inflammation, asthma

## Abstract

Allergic asthma is a chronic pulmonary disorder fundamentally linked to immune dysfunction. Since the immune system begins developing *in utero*, prenatal exposures can affect immune programming and increase risk for diseases such as allergic asthma. Chronic psychosocial stress during pregnancy is one such risk factor, having been associated with increased risk for atopic diseases including allergic asthma in children. To begin to define the underlying causes of the association between maternal stress and allergic airway inflammation in offspring, we developed a mouse model of chronic heightened stress hormone during pregnancy. Continuous oral administration of corticosterone (CORT) to pregnant mice throughout the second half of pregnancy resulted in an ~2-fold increase in circulating hormone in dams with no concomitant increase in fetal circulation, similar to the human condition. To determine how prolonged heightened stress hormone affected allergic immunity in offspring, we induced allergic asthma with house dust mite (HDM) and examined the airway immune response to allergen. Female mice responded to HDM more frequently and had a more robust immune cell response compared to their male counterparts, irrespective of maternal treatment. Male offspring from CORT-treated dams had a greater number of inflammatory cells in the lung in response to HDM compared to males from control dams, while maternal treatment did not affect immune cell numbers in females. Alternatively, maternal CORT caused enhanced goblet cell hyperplasia in female offspring following HDM, an effect that was not observed in male offspring. In summary, prenatal exposure to mild, prolonged heightened stress hormone had sexually dimorphic effects on allergic inflammation in airways of adult offspring.

## Introduction

Allergic asthma is a chronic pulmonary disorder that is typically characterized by T-helper cell type-2 (T_H_2) inflammation, airway hyperresponsiveness, goblet cell hyperplasia, and mucus hypersecretion (1, 2). In patients with asthma, allergen exposure typically drives the polarization and migration of allergen-specific T_H_2 cells to the lungs. These cells mediate the physiological response to allergen by secreting cytokines such as interleukin (IL)-5, which results in eosinophil recruitment, IL-4 and IL-13 secretion, leading to hyperproliferation of goblet cells and increased mucus production (2, 3). While this is the classic response associated with allergic asthma, more severe forms have also been associated with pro-inflammatory T_H_1 or T_H_17 cells, monocytes, and neutrophil recruitment to the lungs (4). The significant heterogeneity in cellular responses and disease severity in the human population demonstrates the complex etiology underlying this allergic airway disease.

Allergic asthma is fundamentally linked to immune dysfunction. The immune system begins developing prenatally and can be significantly influenced by the maternal environment (5, 6). Maternal lifestyle, behaviors and physical exposures influence the *in utero* environment and can affect immune programming at critical windows of time during fetal development (7–11). Some exposures, such as those associated with rural environments, can have beneficial effects on immune maturation and function (12–16). Alternatively, other exposures, such as maternal psychosocial stress, can have adverse effects on immune development, increasing risk for immune-related disorders (17–21).

Indeed, maternal psychosocial stress during pregnancy has been associated with asthma risk in offspring in numerous epidemiological studies (17–21). During times of chronic stress women can experience 2–3-fold increased circulating cortisol (22), the master regulator of the stress response in humans. Importantly, despite the increase in circulating cortisol in the mother, cortisol is typically not heightened in the fetus thanks to placental expression of 11-beta hydroxysteroid dehydrogenase-2 (11β-HSD2), an enzyme that converts cortisol to its inactive form (23, 24). This suggests that, in humans, maternal stress likely affects fetal programming of allergic asthma indirectly, potentially by affecting placental function (25–28).

The link between prenatal maternal stress and allergic asthma in offspring has also been demonstrated in rodents (29) In mice, offspring from “stressed” dams have increased airway hyper-responsiveness and T_H_2 polarization in response to sensitization and challenge with ovalbumin (OVA) compared to control offspring. While these studies have demonstrated that the link between maternal stress and allergic asthma phenotypes exists in rodents, it is unclear how well these models represent the human condition of chronic stress. Moreover, most animal models used to date have utilized tools that cause brief stress-induction over a short time during gestation, thus mimicking acute stress, which is physiologically distinct from the chronic stress exposure associated with asthma risk in humans (29–31). Additionally, significantly elevated levels of stress hormone were also observed in the fetus using an acute stress model (31), which would differ from what is observed in humans. These differences could limit the suitability of these models for more in-depth studies that aim to uncover the underlying mechanism linking chronic maternal stress to asthma risk in humans.

In the present study, we aimed to develop a mouse model to study the effects of chronic heightened stress hormone during pregnancy on fetal programming of allergic asthma. Chronic psychosocial stress is a heterogeneous condition influenced by numerous societal and genetic factors, making it difficult, if not impossible, to naturally recapitulate in rodents. But, while we cannot know what natural situations in rodents, if any, cause the low-grade, chronic stress that negatively affects human health, we can still attempt to mimic the key physiological aspects of chronic stress experienced by pregnant women. Therefore, we administered corticosterone (CORT), the active stress hormone in mice, in drinking water at a concentration that resulted in an ~2-fold increase in maternal circulation, without a concomitant increase in fetal circulation. We then confirmed that oral administration of corticosterone affected allergic asthma phenotypes in offspring, suggesting this could be an important model for future studies aiming to characterize the mechanistic link between chronic maternal stress and asthma risk in humans.

## Materials and Methods

### Animals: Corticosterone Treatment

All animal work was done in accordance with protocols approved by the Michigan State University Institutional Animal Care and Use Committee. Eight-to-twelve weeks old primiparous C57Bl/6J mice (Jackson Labs, Bar Harbor, ME) were housed under 12-h light and dark cycles with *ad libitum* access to food and water. Females were time-mated to males of proven fertility and the presence of a copulation plug was denoted as embryonic day 0.5 (E0.5). On E12.5 of pregnancy, females received oral administration of vehicle (VEH) or corticosterone (CORT) (Sigma-Aldrich, St. Louis, MO) in distilled water. Corticosterone was dissolved in 25% (2-hydroxypropyl)-β-cyclodextrin (2-HβC) (Sigma-Aldrich, St. Louis, MO) to a concentration of 10 mg/ml, then diluted to a working concentration of 50 μg/mL. 2-HβC is a carrier molecule that increases the solubility of CORT without adverse effects during pregnancy in rodents (32). Vehicle treatment contained 2-HβC at a final concentration of 0.1875%. Treatment was refreshed every 3 days. To assess CORT levels during pregnancy, pregnant dams were euthanized on E18.5 and maternal blood was collected via cardiac puncture at 7:30 p.m., the beginning of the dark cycle. Trunk blood was collected and pooled from each fetus in a litter. Once the CORT regiment was established it was used to assess the effects of heightened maternal CORT during pregnancy on allergic airway inflammation in offspring. Dams received CORT treatment, as described at E12.5, and were removed from CORT treatment following birth of their pups.

### Animals: House Dust Mite Treatment

At 4 weeks of age offspring were weaned, separated by sex, and began intranasal installments with house dust mite (HDM) or saline. Offspring were sensitized with HDM (Greer Labs, Lenoir, NC, lot # 253983) at a concentration of 135 μg/mL of the major allergen Der p 1, in 30 μl saline with three intranasal installments (d1, d4, d7) and then were challenged with a 2-fold higher dose (270 μg/mL Der p 1) on d21 (33, 34). Endotoxin values for HDM lot# 253983 were 1126 EU/μg Der p 1 and therefore 135 μg/mL of Der p 1 contained 4.05 EU endotoxin, and 270 μg/mL Der p 1 contained 8.1 EU endotoxin.

### Bronchoalveolar Lavage

Offspring were euthanized 48 h post-challenge and lungs were lavaged with 0.8 mL ice-cold 1X PBS supplemented with 0.2 mM EDTA (bronchoalveolar lavage fluid or BAL). An aliquot of BAL was applied to a hemocytometer and total cell numbers were calculated. An aliquot of BAL was then applied to slides using the Shandon Cytospin, fixed with methanol, and stained with modified Wright-Giemsa stain (Sigma-Aldrich). Two individuals, blinded to treatment, counted 200 cells/slide and identified cells as macrophages, eosinophils, neutrophils or lymphocytes. Total cell numbers were then extrapolated for each cell type. The remaining BAL was centrifuged and the supernatant was stored at −80°C prior to cytokine analysis.

### Lung Histology and PAS Staining

Lungs were inflated with 4% paraformaldehyde at a pressure of 30 cm using a 22- gauge catheter, and were fixed overnight at room temperature. After fixation, lung tissues were dehydrated in a graded series of ethanol solution, embedded in paraffin, and 6 μm transverse sections were obtained. The lung sections were stained with hematoxylin and Eosin (H&E) or Periodic acid-Schiff (PAS)-hematoxylin (Sigma-Aldrich, St. Louis, MO) to examine histological signs of inflammation including immune cell infiltration and goblet cell hyperplasia, respectively. Goblet cell hyperplasia was quantified using methods previously described by Padrid et al. (35) with some modifications. Briefly, 10 fields per lung were randomly selected and histological modifications in goblet cells were scored according to the percentage of PAS-positive cells lining the bronchoalveolar space: grade 0: <0.5%; grade 1: 0–25%, grade 2: 25–50%, grade 3: 50–75%, grade 4: >75%. The mean scores of positive PAS cells in each mouse were calculated.

### ELISA and Luminex

Circulating levels of CORT in pregnant dams and fetuses was measured using the corticosterone competitive ELISA assay (IBL/Tecan, Mannedorf, Switzerland) per manufacturer's protocol. Maternal serum was diluted 1:100 and fetal serum was diluted 1:5 prior to assay. Total IgE concentrations were determined using the ELISA MAX mouse IgE ELISA kit (BioLegend, San Diego, CA) according to the manufacturer's protocol. Serum was diluted 1:25 prior to assay. Cytokine concentrations in BAL were determined using a 23-plex cytokine assay (BioRad, Hercules, CA) per manufacturers instructions and analyzed with the LUMINEX 200 (LUMINEX, Austin, TX). Cytokines included in this assay were: Eotaxin, G-CSF, GM-CSF, IFNg, IL-1a, IL-Ib, IL-2, IL-3, IL-4, IL-5 IL-6, IL-9, IL-10, IL-12p40, IL-12p70, IL-13, IL-17, KC, MCP-1, MIP-1a, MIP-1b, RANTES, and TNFa.

### Statistical Analysis

Maternal and fetal circulating CORT concentration means were compared between VEH- and CORT-treated dams using Student *T*-test and results were reported as means ± SEM. The HDM responder frequency of male and female offspring was compared using Fisher's Exact Test. Male offspring that did not respond to HDM were not used in further analysis. Cytokine mean concentrations in BAL from HDM-treated offspring were compared between offspring of VEH- and CORT-treated dams using Student *T*-test and results were reported as means ± SEM. A two-way ANOVA was used to assess the effect of offspring sex, HDM treatment, and the interaction of sex and HDM on BAL immune cells and lung histology (PAS), while multiple comparisons were made using the Tukey *post-hoc* test. A two-way ANOVA was used to assess the effect of maternal treatment, HDM treatment, and the interaction of maternal treatment and HDM on BAL immune cells and lung histology (PAS), while multiple comparisons were made using the Tukey *post-hoc* test. When analyzing the effect of maternal treatment on offspring responses, since the dam was the experimental unit, only one offspring/treatment was used from each litter to ensure equal variance and sample independence. All data was normal and significance was reported according to *p*-value, with *p* ≤ 0.05 considered statistically significant. Analyses were performed using Prism software. Sample numbers for each experimental group are reported for each experiment within the figure legends.

## Results

### Mouse Model of Chronic Heightened Stress Hormone During Pregnancy

**Figure 1:**
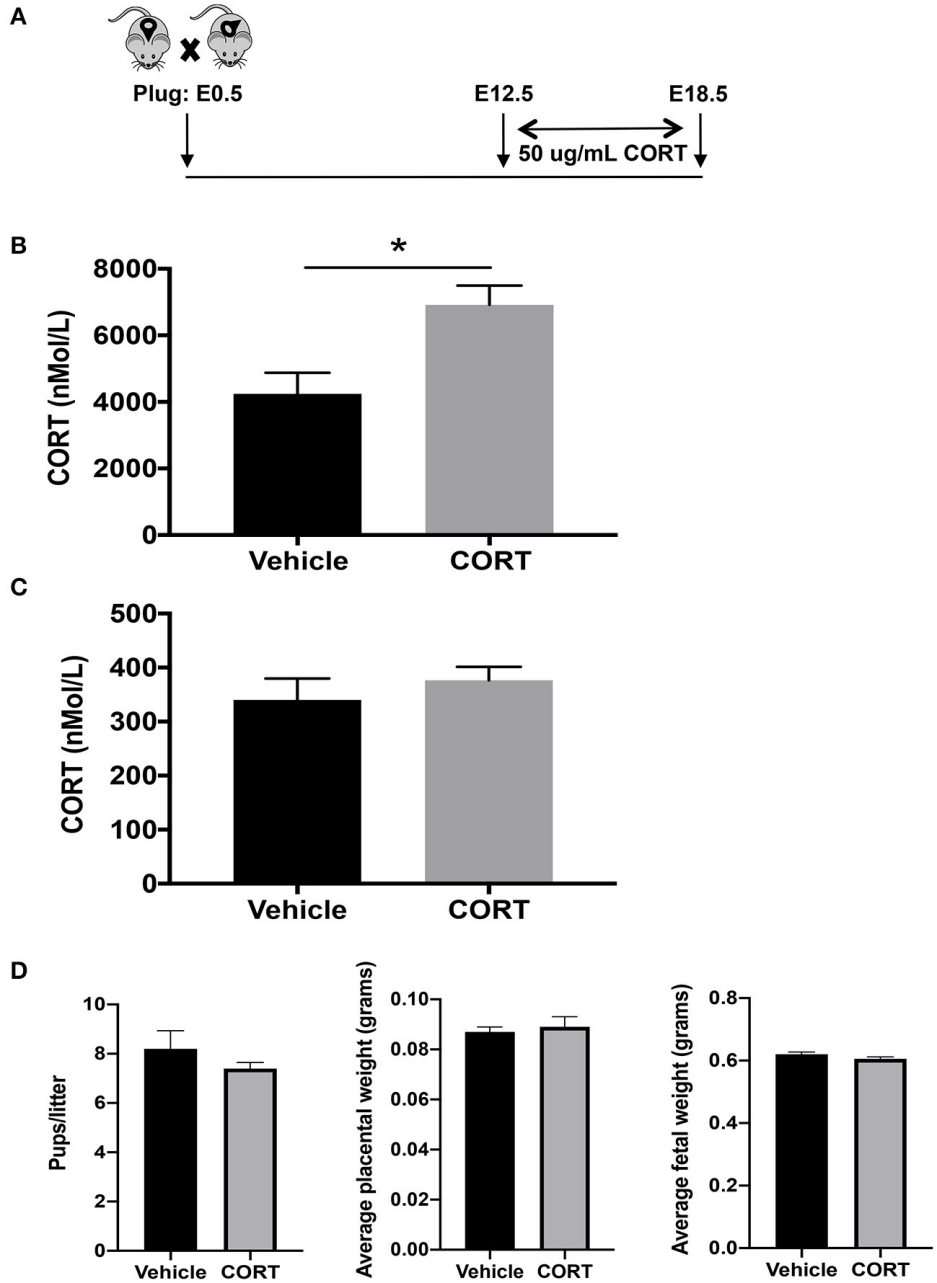
Mouse model of chronic heightened stress hormone during pregnancy. **(A)** Schematic representation of maternal corticosterone (CORT) treatment during pregnancy. Male and female mice were housed together overnight; females with a copulation plug were identified and this was considered embryonic day E 0.5. Pregnant females began oral treatment with 50 μg/mL CORT or vehicle in drinking water at E12.5 until the end of pregnancy on E19.5. Blood was collected from CORT and vehicle-treated dams **(B)** and fetuses of CORT and vehicle-treated dams **(C)** at 7:30 p.m. on E17.5 and CORT concentrations were analyzed using ELISA. Circulating CORT concentrations reported as nmol/L in serum. **(D)** Maternal treatment did not affect birth outcomes including length of gestation, number of pups, or weights of placentas or pups (*p < 0.05, *n* = 5–7).

We first developed a mouse model to study how chronic, elevated maternal CORT during pregnancy affects allergic airway inflammation in future offspring. We delivered CORT, the primary stress hormone in mice, to pregnant dams in the drinking water from pregnancy day 12.5 and continued until day 19.5, the last day of gestation (Figure 1A). Specifically, CORT was dissolved in 25% 2-HβC and diluted to a working concentration of 50 μg/mL in water (Figure 1A). This treatment protocol raised circulating CORT concentrations ~2-fold in the circulation of pregnant dams compared to vehicle-treated dams (Figure 1B), thus mimicking the level of increased cortisol during times of reported chronic stress in humans. Importantly, this treatment did not increase the concentration of CORT in fetal circulation (Figure 1C). In addition, treatment did not affect birth outcomes including length of gestation, number of pups, or weights of placentas or pups (Figure 1D).

### Female Mice Have a More Robust Pulmonary Inflammatory Response to HDM Compared to Males

**Figure 2:**
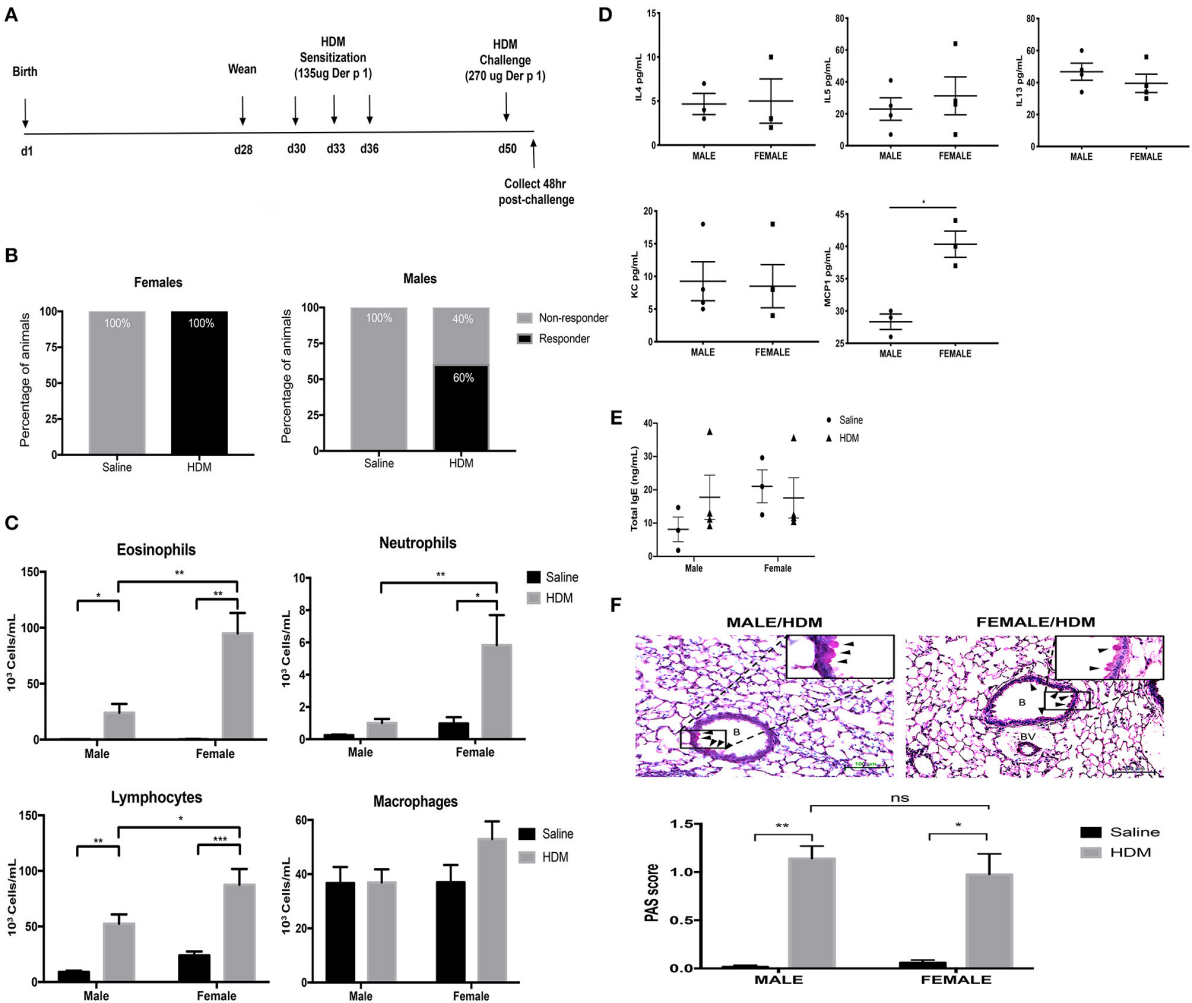
Characterization of pulmonary immune cells, cytokines, and histopathology in male and female mice. **(A)** Schematic representation of protocol for induction of allergic asthma in mice. At 28 days of age offspring were weaned, separated by sex, and began intranasal instillation with house dust mite (HDM) or saline. Offspring were sensitized with three intranasal installments of HDM consisting of 135 μg of the major allergen Der p 1 (d30, d33, d36), were challenged with a 2-fold higher dose on d50, and tissue was collected 48 h post-challenge. **(B)** The percentage of male and female responders to HDM, defined as 10% or greater eosinophil accumulation in the lung; female responders, *n* = 17/17 (100%), male responders, *n* = 9/16 (60%). **(C)** Immune cells were quantified, total eosinophils (*p = 0.002, **p < 0.0001), neutrophils (*p = 0.003, **p = 0.0014), lymphocytes (*p = 0.03, **p = 0.0008, ***p < 0.0001), and alveolar macrophages in bronchoalveolar lavage fluid (BAL) of male (*n* = 8–10) and female (*n* = 7–10) responders. **(D)** Cytokine and chemokine concentrations in BAL of male and female HDM-treated mice. Interleukin-4 (IL-4), interleukin-5 (IL-5), interleukin-13 (IL-13), and monocyte chemoattractant protein-1 (MCP-1) concentrations are reported as pg/mL in BALF (*p < 0.05, *n* = 4). **(E)** Total IgE in serum of male and female offspring treated with saline (circle) and HDM (triangle), with concentrations reported as ng/mL (*n* = 3–5). **(F)** Representative light photomicrographs of lung tissue sections from male and female mice stained with Periodic acid-Schiff (PAS)-hematoxylin. B, bronchiolar airspace; BV, blood vessel; arrows, PAS+ cells; scale bar: 100 μm. Goblet cell hyperplasia was quantified by scoring histological sections based on the percentage of PAS-positive cells lining the bronchoalveolar space: grade 0: <0.5%; grade 1: 0–25%, grade 2: 25–50%, grade 3: 50–75%, grade 4: >75%. The mean PAS scores were compared (*p = 0.0006, **p < 0.0001) (*n* = 5–7).

Before determining how maternal CORT treatment affected the allergic airway inflammatory response in offspring, we first had to establish the baseline response for our allergic asthma induction protocol using healthy offspring from non-treated dams. Animals underwent trifold sensitizations with 10 μg HDM (containing 135 μg Der P 1) and were challenged with 20 μg HDM (containing 270 μg Der P 1) (Figure 2A). All female offspring (*n* = 17/17) amassed an allergic inflammatory response to HDM, defined by 10% or greater eosinophil accumulation in the lung. Conversely, only 60% of male offspring developed allergic inflammation in response to HDM (*n* = 9/16), a lower frequency than females (Figure 2B) (Fisher's exact, *p* = 0.005). The immune response to HDM was also more robust in females compared to that of the male responders. While HDM-treatment significantly increased eosinophils in lungs of both males and females compared to saline-treated controls, there was a 3-fold increase in eosinophil response in HDM-treated females compared to HDM-treated males (two-way ANOVA, eosinophils: effect of sex, *p* < 0.0001, effect of HDM, *p* < 0.0001, interaction, *p* < 0.0001; Tukey *post-hoc*: Males^saline vs.HDM^, *p* = 0.002; Females^saline vs.HDM^, *p* < 0.0001, HDM^male vs.Female^, *p* < 0.0001) (Figure 2C). Only females had a significant increase in lung neutrophils (two-way ANOVA, neutrophils: effect of sex, *p* < 0.0001, effect of HDM, *p* < 0.0001, interaction, *p* = 0.0005; Tukey *post-hoc*: Female^saline vs.HDM^, *p* = 0.0003, HDM^male vs.Female^, *p* = 0.0014), Females had greater lymphocyte recruitment to the lung following HDM treatment compared to males (two-way ANOVA, lymphocytes: effect of sex, *p* < 0.01, effect of HDM, *p* < 0.0001, interaction, *p* = 0.10; Tukey *post-hoc*: Male^saline vs.HDM^, *p* = 0.0008; Female^saline vs.HDM^, *p* < 0.0001, HDM^male vs.Female^, *p* = 0.03). The number of alveolar macrophages was not affected by sex or treatment (Figure 2C).

The cytokine and chemokine profile in BAL was also compared between HDM-treated male and female offspring. Three T_H_2 cytokines with established roles in allergic inflammation, IL-4, IL-5, and IL-13, were identified in BAL from HDM-treated males and females, but concentrations were not affected by sex (Student's *t*-test: IL-4 (*p* = 0.5), IL-5 (*p* = 0.2), and IL-13 (*p* = 0.4) (Figure 2D). The only soluble immune factor that was different between males and females was the monocyte chemoattractant protein-1 (MCP-1), a key mediator of inflammatory cell recruitment to the lung (36). The concentration of MCP-1 was greater in BAL of HDM-treated females compared to male counterparts (Student *t*-test, *p* < 0.004) (Figure 2D). Other cytokines and chemokines analyzed, but with no significant changes, included Eotaxin, G-CSF, GM-CSF, IFNg, IL-1a, IL-Ib, IL-2, IL-3, IL-6, IL-9, IL-10, IL-12p40, IL-12p70, IL-17, KC, MIP-1a, MIP-1b, RANTES, and TNFa.

### Goblet Cell Hyperplasia and Total Serum IgE Following HDM Treatment Is Similar in Male and Female Mice

In addition to immune activation, allergic airway inflammation is associated with goblet cell hyperplasia and mucus hypersecretion, which contribute to airway remodeling and can significantly impair lung function (33). Histological modifications in mucus-containing goblet cells were scored according to the percentage of PAS+ peribronchiolar cells lining the bronchoalveolar space using ten randomly selected fields per lung. Interestingly, while females had enhanced immune cell recruitment to the lung, males and females had a similar increase in PAS+ peribronchiolar cells in response to HDM (Figure 2E). The mean scores of PAS+ cells were significantly greater in lungs from HDM-treated mice compared to saline-treated mice, but there was no effect of sex (two-way ANOVA, PAS score: Effect of HDM, *p* < 0.0001, effect of sex, *p* = 0.60 interaction, *p* = 0.37; Tukey *post-hoc*: Male^saline vs.HDM^, *p* < 0.0001 Female^saline vs.HDM^, *p* = 0.0006, HDM^male vs.female^, *p* = 0.76) (Figure 2F). Total serum IgE levels were not affected by HDM or sex of the offspring (Figure 2E).

### Maternal CORT Enhances Airway Inflammation in Male Offspring

**Figure 3:**
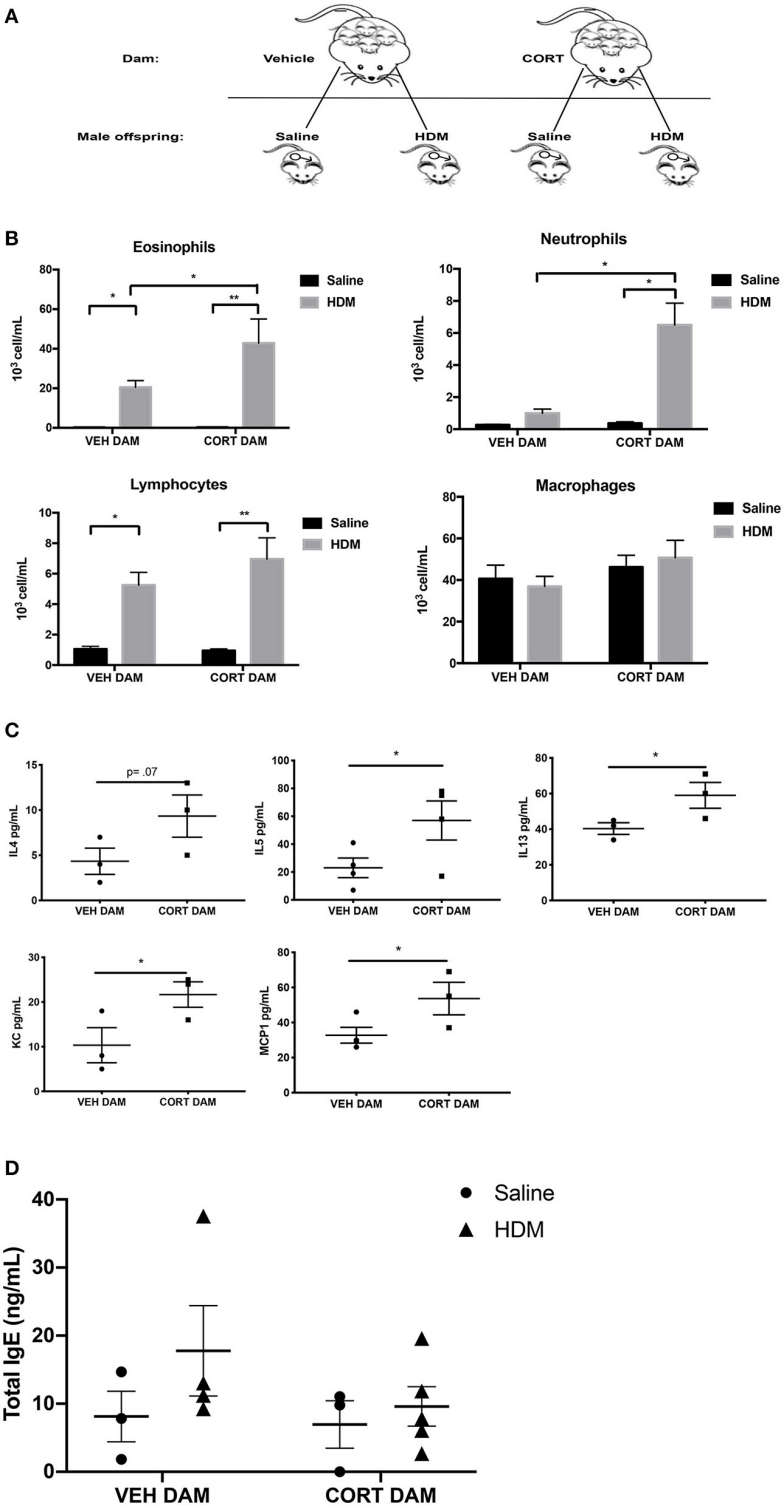
Pulmonary immune cells, cytokines, and total serum IgE in male offspring from vehicle and CORT-treated dams. **(A)** Schematic representation of dam and offspring treatments. Pregnant females received corticosterone (CORT) or vehicle in drinking water at E12.5 until the end of pregnancy. Male offspring from CORT and vehicle treated dams were treated with saline or HDM consisting of 135 μg of the major allergen Der p 1. **(B)** Immune cells were quantified, total eosinophils (*p = 0.02, **p < 0.0001), neutrophils (*p < 0.0001), lymphocytes (*p = 0.0009, **p < 0.0001), and alveolar macrophages in BAL of male offspring from vehicle or CORT-treated dams, treated with HDM or saline (*n* = 6–8). **(C)** Cytokine and chemokine concentrations in BALF from HDM-treated male offspring from vehicle and CORT-treated dams. Interleukin-4 (IL-4), interleukin-5 (IL-5), interleukin-13 (IL-13), keratinocyte chemoattractant (KC), and monocyte chemoattractant protein-1 (MCP-1) concentrations are reported as pg/mL in BALF. **(D)** Total IgE in serum of male offspring treated with saline (circle) and HDM (triangle), with concentrations reported as ng/mL (*p < 0.05, *n* = 3–5).

Next, allergic airway inflammation was compared between male offspring of vehicle (VEH) and CORT-treated dams following HDM sensitization and challenge (Figure 2A). Offspring from VEH- and CORT-treated dams were separated at weaning and received saline or HDM treatment (Figure 3A). Response rates to HDM (>10% eosinophil infiltration in BALF) did not differ in males born to CORT or vehicle treated dams and males that did not display airway eosinophilia were excluded from further analysis. Males from CORT-treated dams had significantly more eosinophils recruited to the lung following HDM treatment than males born to VEH-treated dams (Figure 3B; two-way ANOVA, eosinophils: Effect of maternal treatment, *p* = 0.03, effect of HDM treatment, *p* < 0.0001, interaction, *p* = 0.03; Tukey *post-hoc*: VEH(DAM)^saline vs.HDM^, *p* = 0.02, CORT(DAM)^saline vs.HDM^, *p* < 0.0001, HDM^VEH vs.CORT^, *p* = 0.03). Male offspring from CORT-treated dams also had significantly more neutrophils in the lung following HDM challenge compared to males from VEH-treated dams (Figure 3B; two-way ANOVA, neutrophils: Effect of maternal treatment, *p* < 0.0001, effect of HDM treatment, *p* < 0.0001, interaction, *p* < 0.0001; Tukey *post-hoc*: VEH(DAM)^saline vs. HDM^, *p* = 0.75, CORT(DAM)^saline vs.HDM^, *p* < 0.0001, HDM^VEH vs. CORT^, *p* < 0.0001). Male offspring from VEH- and CORT-treated dams had similar, and significant, lymphocyte recruitment to the lung following HDM treatment (two-way ANOVA, lymphocytes: Effect of maternal treatment, *p* = 0.27, effect of HDM treatment, *p* < 0.0001, interaction, *p* = 0.22; Tukey *post-hoc*: VEH(DAM)^saline vs.HDM^, *p* = 0.0009, CORT(DAM)^saline vs.HDM^, *p* < 0.0001, HDM^VEH vs. CORT^, *p* = 0.38). The number of alveolar macrophages was not affected by sex or treatment (Figure 3B).

We characterized the cytokine and chemokine profile in BAL of HDM-treated male offspring from VEH- and CORT-treated dams. There were significantly higher concentrations of IL-5 (*p* = 0.03), IL-13 (*p* = 0.05), keratinocyte chemoattractant (KC), an important regulator of neutrophil recruitment (*p* = 0.04), and MCP-1 (*p* = 0.04) in offspring from CORT-treated dams compared to VEH-treated dams (Student *t*-test) (Figure 3C). There was a tendency for higher concentrations of IL-4 in the BAL of males from CORT-treated dams, but this did not reach significance (*p* = 0.07) (Figure 3C). Other cytokines and chemokines analyzed, but with no significant changes, included Eotaxin, G-CSF, GM-CSF, IFNg, IL-1a, IL-Ib, IL-2, IL-3, IL-6, IL-9, IL-10, IL-12p40, IL-12p70, IL-17, MIP-1a, MIP-1b, RANTES, and TNFa.). In addition, total serum IgE levels were not affected by HDM or maternal treatment in male offspring (Figure 3D).

### Maternal CORT Does Not Affect Goblet Cell Hyperplasia in Male Offspring

**Figure 4:**
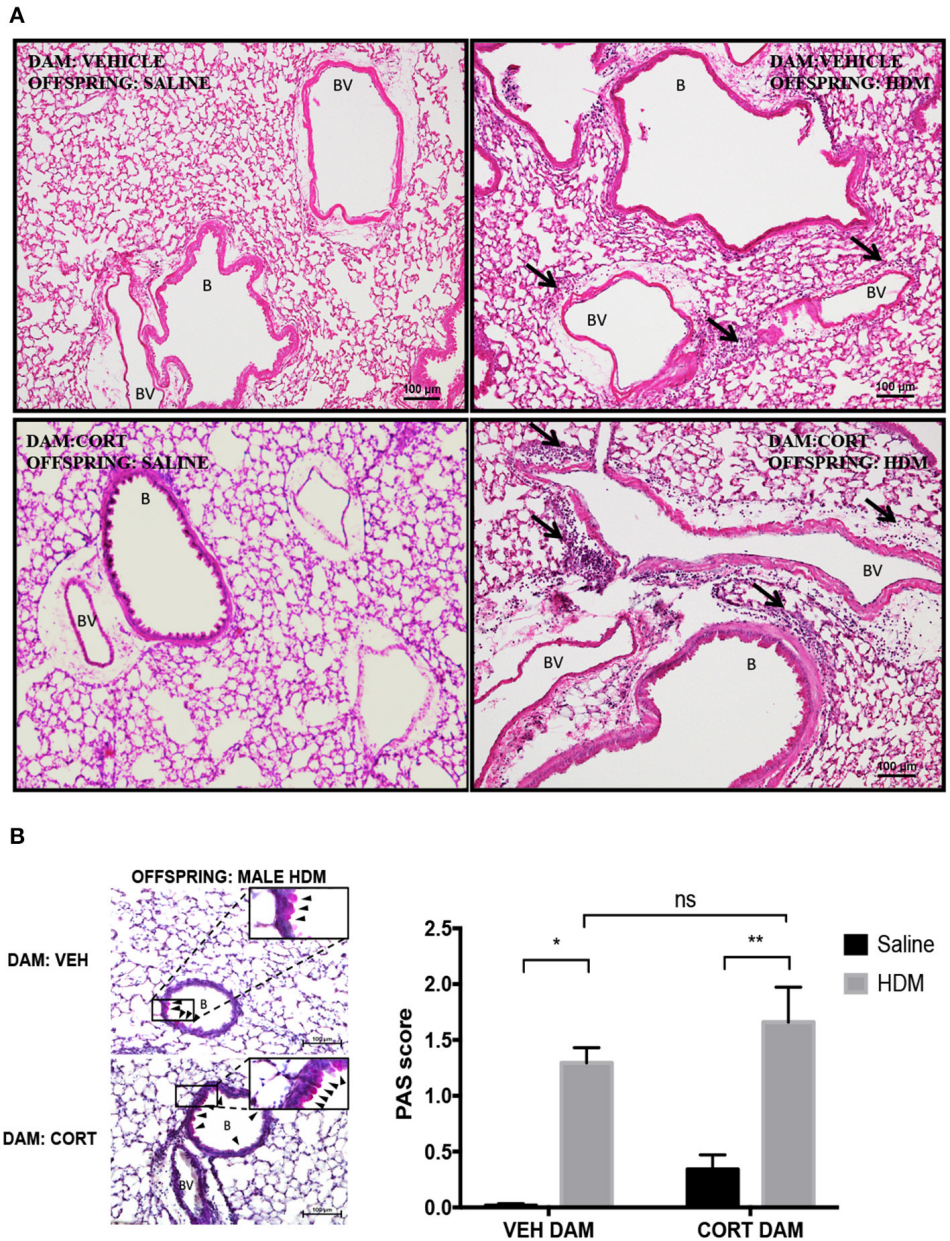
Characterization of pulmonary histopathology in male offspring from vehicle and CORT-treated dams. **(A)** Representative light photomicrographs of lung tissue sections from male offspring of vehicle and CORT-treated dams stained with hematoxylin and Eosin. B, bronchiolar airspace; BV, blood vessel; arrows, immune cell infiltration. **(B)** Goblet cell hyperplasia was quantified by scoring histological sections based on the percentage of PAS-positive cells lining the bronchoalveolar space: grade 0: <0.5%; grade 1: 0–25%, grade 2: 25–50%, grade 3: 50–75%, grade 4: >75%. B: bronchiolar airspace, BV: blood vessel, arrows: PAS+ cells, scale bar: 100 μm. The mean PAS scores were compared (*p = 0.002, **p = 0.0002) (*n* = 3–5).

We next examined pulmonary histology in lung tissue stained with H & E and PAS-hematoxylin to examine immune cell infiltration and goblet cell hyperplasia in male offspring from vehicle and CORT-treated dams. Moderate peribronchiolar and perivascular immune cell infiltration into lung tissue was apparent in offspring treated with HDM (Figure 4A), and was more robust in offspring from CORT-treated dams in accordance with the cytospin results (Figure 3B). To quantify goblet cell metaplasia, the percentage of PAS+ peribronchiolar cells lining the bronchoalveolar space were determined and used to produce quantifiable mean scores. While the mean scores of PAS+ cells were significantly increased by HDM treatment in male offspring, the PAS scores were not affected by maternal CORT treatment (two-way ANOVA, PAS score: Effect of maternal CORT, *p* = 0.08, effect of HDM, *p* < 0.0001, interaction, *p* = 0.91; Tukey *post-hoc*: VEH(DAM)^saline vs.HDM^, *p* = 0.0002, CORT(DAM)^saline vs.HDM^, *p* = 0.002, HDM^VEHDAM vs.CORTDAM^, *p* = 0.44) (Figure 4B).

### Maternal CORT Affects Goblet Cell Metaplasia, but Not Immune Cell Recruitment, in Female Offspring

**Figure 5:**
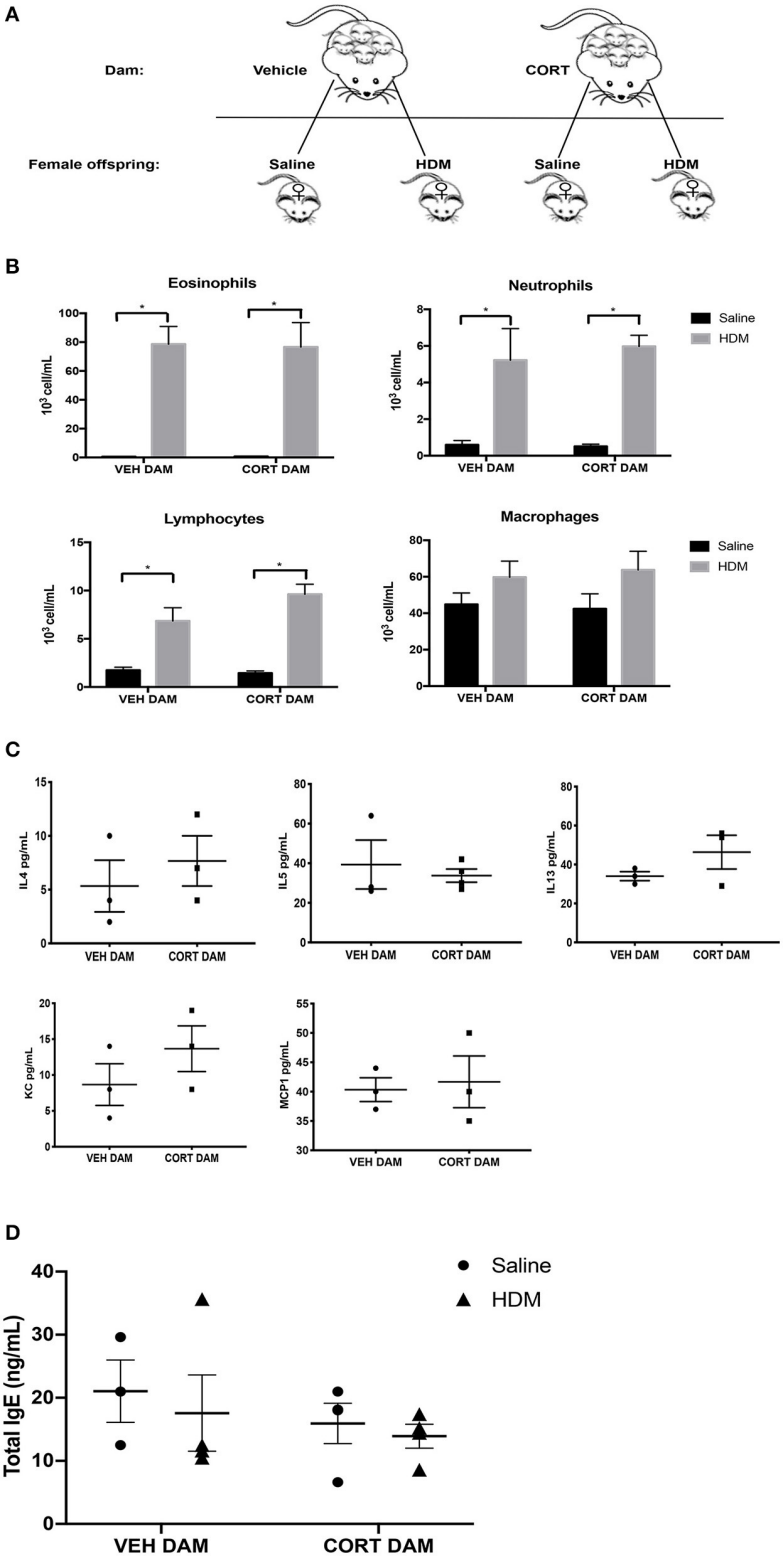
Pulmonary immune cells, cytokines and total serum IgE in female offspring from vehicle and CORT-treated dams. **(A)** Schematic representation of dam and offspring treatments. Pregnant females received corticosterone (CORT) or vehicle in g water at E12.5 until the end of pregnancy. Female offspring from CORT and vehicle treated dams were treated with saline or HDM consisting of 135 μg of the major allergen Der p 1. **(B)** Immune cells were quantified, total number of eosinophils, neutrophils, lymphocytes, and alveolar macrophages in BAL of female offspring from vehicle or CORT-treated dams, treated with HDM or saline (*n* = 5–9). **(C)** Cytokine and chemokine concentrations in BALF were compared between HDM-treated female offspring from vehicle and CORT-treated dams. Interleukin-4, IL-5, IL-13, KC, and MCP-1 concentrations are reported as pg/mL in BALF. **(D)** Total IgE in serum of female offspring treated with saline (circle) and HDM (triangle), with concentrations reported as ng/mL (*n* = 4). * *p* < 0.0001.

Next, we examined the effect of maternal CORT treatment on allergic airway inflammation in female offspring (Figure 5A). Females underwent HDM sensitization and challenge using the protocol described in Figure 2A, and then we analyzed immune cell recruitment, cytokines and PAS+ cells in the lungs. While female offspring had significant increases in eosinophils, neutrophils and lymphocytes following HDM, the immune cell response was not affected by maternal CORT treatment (two-way ANOVA, eosinophils: Effect of maternal CORT, *p* = 0.91, effect of HDM, *p* < 0.0001, interaction, *p* = 0.89; two-way ANOVA, neutrophils: Effect of maternal CORT, *p* = 0.73, effect of HDM, *p* < 0.0001, interaction, *p* = 0.66; two-way ANOVA, lymphocytes: Effect of maternal CORT, *p* = 0.18, effect of HDM, *p* < 0.0001, interaction, *p* = 0.10) (Figure 5B). Similarly, maternal CORT treatment did not affect the cytokine concentrations in lungs following HDM challenge (Student *t*-test: IL-4, *p* = 0.26, IL-5, *p* = 0.32, IL-13, *p* = 0.12, KC, *p* = 0.16, MCP-1, *p* = 0.40) (Figure 5C). In addition, total serum IgE levels were not affected by HDM or maternal treatment in female offspring (Figure 5D).

**Figure 6:**
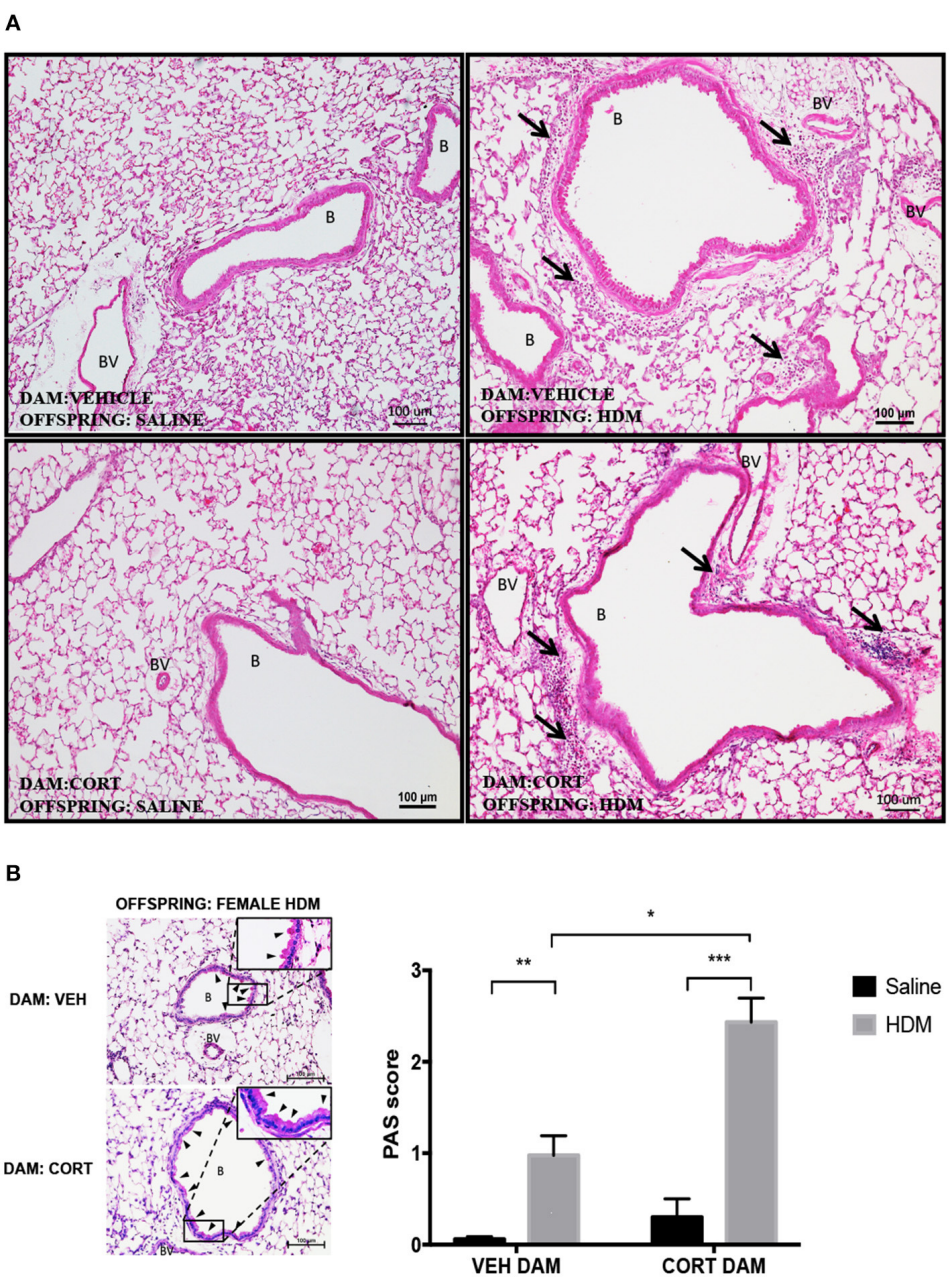
Characterization of pulmonary histopathology in female offspring from vehicle and CORT-treated dams. **(A)** Representative light photomicrographs of lung tissue sections from male offspring of vehicle and CORT-treated dams stained with hematoxylin and eosin. B, bronchiolar airspace; BV, blood vessel; arrows, immune cell infiltration. **(B)** Goblet cell hyperplasia was quantified by scoring histological sections based on the percentage of PAS-positive cells lining the bronchoalveolar space: grade 0: <0.5%; grade 1: 0–25%, grade 2: 25–50%, grade 3: 50–75%, grade 4: >75%. B, bronchiolar airspace; BV, blood vessel; arrows, PAS+ cells, scale bar: 100 μm. The mean PAS scores were compared (*p = 0.01, **p = 0.0003, ***p < 0.0001) (*n* = 5–6).

Again we examined pulmonary histology in lung tissue stained with H & E and PAS-hematoxylin to examine immune cell infiltration and goblet cell hyperplasia in female offspring from vehicle and CORT-treated dams. Moderate peribronchiolar and perivascular immune cell infiltration into lung tissue was apparent in offspring treated with HDM (Figure 6A), and was not affected by maternal treatment in accordance with the cytospin results (Figure 5B). Interestingly, unlike males, goblet cell hyperplasia was affected by maternal CORT treatment (Figure 6B). The mean PAS+ scores were significantly greater in female offspring of CORT-treated dams compared to those from VEH-treated dams following HDM challenge (two-way ANOVA, PAS score: Effect of maternal CORT, *p* = 0.0003, effect of HDM, *p* < 0.0001, interaction, *p* = 0.005; Tukey *post-hoc*: VEH(DAM)^saline vs.HDM^, *p* = 0.01, CORT(DAM)^saline vs.HDM^, *p* < 0.0001, HDM^VEHDAM vs. CORTDAM^, *p* = 0.0003) (Figure 6B).

## Discussion

In this study, we sought to develop a new mouse model of chronic heightened stress hormone during pregnancy to study the effects of prenatal chronic stress hormone exposure on the programming of allergic airway inflammation in offspring. We determined that continuous oral administration of CORT to pregnant mice throughout the second half of pregnancy resulted in an ~2-fold increase in circulating hormone in dams with no concomitant increase in fetal circulation, and with no apparent effect on overall health of the dam, length of gestation or litter size. We next tested the effects of maternal CORT on allergic airway inflammation in offspring following sensitization with house dust mite (HDM). Interestingly, to the best of our knowledge, we are the first to report that chronic, heightened CORT in the dam had sexually dimorphic effects on allergic airway inflammation in offspring. Specifically, male offspring from CORT-treated dams had a greater number of inflammatory cells in the lung in response to HDM compared to males from control dams, while immune cell numbers were not changed in female offspring. Alternatively, maternal CORT caused enhanced goblet cell hyperplasia in female offspring following HDM, an effect not observed in male offspring. These results suggest that this model could be a useful tool for better understanding the effects of chronic stress on fetal immune development and postnatal immune outcomes.

The first goal of this study was to develop a mouse model that could be used to study the physiological effects of chronic stress hormone exposure on fetal development and postnatal immune function. The association between maternal stress and atopic disease in offspring is well-documented, but the mechanistic link between exposure and outcome remains unknown. Rodents have been useful models for mechanistic studies, but previous studies utilized acute stress, which is physiologically distinct from the chronic stress condition associated with asthma risk in humans (29, 31). Unfortunately, we cannot know what a rodent perceives as a “chronic stressor,” but we can recapitulate some key physiological aspects of chronic stress that have been documented in women. For example, it is known that the stress hormone, cortisol, is the master regulator of the physiological stress response and pregnant women with chronic stress maintain a low-grade (~2-fold) elevation in circulating cortisol over time (22). There is also evidence that heightened CORT is a primary upstream regulator of the negative effects on fetal programming in rodents. For example, inhibiting CORT synthesis in pregnant mice prior to stress treatment abrogates the stress-associated changes in airway inflammation in offspring (31). Importantly, these data suggest that maternal CORT is the upstream mediator of stress-associated, *in utero* programming of allergic diseases. Therefore, we hypothesized that a mouse model that maintained a chronic 2-fold elevation of stress hormone in the dam during pregnancy would mimic many of the key physiological aspects of chronic stress that affect fetal immune development and postnatal outcomes. Furthermore, in humans the fetus is typically buffered from fluctuations in maternal stress hormone because the placenta expresses the enzyme 11β-HSD2, which converts cortisol to its inactive metabolite (24). This means that, in humans, chronic maternal stress has indirect effects on the fetus, likely through stress-associated changes in placental function. Therefore, a good model would not only mimic the chronic elevation of stress hormone in the dam, but would not elevate stress hormone in the fetus, which would have direct effects on fetal development that would likely differ from the indirect effects of maternal stress in human pregnancy (22, 23). To achieve this delicate balance, we tested numerous CORT formulations, varying the concentration of CORT and the vehicle of delivery. CORT, a lipophilic hormone, is commonly dissolved in polar solvents, such as methanol or ethanol. We found that using ethanol as the vehicle caused significant wasting and loss of pregnancy. Thus, we adopted a carrier molecule, 2-HβC to increase CORT solubility in drinking water. When delivered in 2-HβC, 50 μg/mL CORT treatment resulted in significant increases in maternal circulating CORT levels without similar changes in fetal CORT levels. Additionally, neither 2-HβC nor CORT affected the health of the pregnancy.

Another goal of this study was to examine sex as a biological variable in the prenatal programming of allergic immune function by stress hormone. Epidemiological studies examining the effect of maternal stress on atopic disease in offspring have conflicting results (12, 20, 37–42). Lee et al. (38) examined 765 mother-child dyads and found an association between prenatal stress and asthma risk in 6 year old boys, while postnatal stress was associated with asthma in 6 years old girls, tendencies that were also observed in another cohort study of Mexican children by Rosa et al. (39), Lee et al. (38). A prospective study of children *in utero* during a severe ice storm in Quebec found that prenatal exposure to this stressful event was associated with increased risk for atopic diseases in girls (20), while other studies found an association between prenatal stress and atopic disease in male and female children (21, 37, 40). These differences suggest that both sexes are likely susceptible to prenatal programming of atopic diseases, but the mechanisms and/or timing of susceptibility could be sexually dimorphic. Our results also suggest that maternal stress affects allergic phentoypes in both sexes, but through divergent mechanisms. In our study, prenatal stress exposure caused enhanced goblet cell metaplasia in the lung of female offspring, while males had enhanced infiltration of immune cells, TH2 cytokines and chemokines associated with monocyte and neutrophil recruitment. While both phenotypes are associated with allergic inflammation, and are regulated by many shared signals, the outcomes are distinct. The differential regulation of these responses in males in females could be the result of sex differences in lung maturation. The male lung matures more slowly compared to the female and it has been proposed that the more mature female fetal lung is more susceptible to the influence of prenatal stressors (6). It could be that CORT treatment affects structural development of the female lung, specifically, resulting in more Clara or ciliated cells, which are the epithelial cell types that convert into mucus-producing goblet cells in response to HDM. The sex differences in the effect of CORT could also be due to differences in immune development. Immune function is less mature in males at birth, with male neonates being more likely to suffer respiratory infections and male children more likely to suffer atopic disease compared to female children (6). The differences in developmental trajectories could affect the impact that CORT treatment has on immune programming. Finally, the placenta is, itself, sexually dimorphic in form and function, and is especially divergent in its response and adaptation to *in utero* exposures (43, 44). Indeed, sex differences in the placental response to stress has been well-documented by others, and these differences are proposed to play an important role in sexually dimorphic programming of development (6, 43). The sex-differences in the placental response to stress are thought to contribute the increased risk for adverse neurodevelopmental programming in males (45), and others have proposed that sex-differences in the placental response to progesterone levels might affect programming of asthma in offspring (46). The interaction of sexually dimorphic organ development with sex differences in placental functions will likely need to be considered in future studies in order to define the mechanisms underlying sex-specific developmental programming.

One of the limitations of this study is the absence of measurements of airway hyperresponsiveness and changes in serum IgE. Our study utilized the allergic asthma induction protocol reported in a study by Brandenberger et al., which reported in response to HDM, moderate immune cell infiltration into airways, increased TH2 cytokines in BAL and increased airway hypersensitivity in response to methacholine. Brandenberger et al., also reported no increase in total serum IgE in response to HDM, similar to our findings. Asthma endotypes are often clustered TH2 high or TH2 low, based on the presence or absence of eosinophils and IL-4, IL-5 and IL-13 cytokines in BAL, all of which we found increased following HDM challenge. Alternatively, while heightened serum IgE is certainly a hallmark of TH2 immune responses, it is not strongly predictive of TH2 signatures in tissues or asthma development in patients (2). Furthermore, it was previously demonstrated that asthma induction protocols require at least 4 weeks of HDM sensitization to induce heightened IgE in serum, which explains why our shorter induction protocol did not result in increased serum IgE (47). Therefore, our results are in accordance with the immune-related phenotypes that were previously characterized (33), and while we did not perform methacholine sensitivity assays, the inflammatory profile observed is suggestive of an allergic asthma phenotype in the lung. Alternatively, because we did not identify asthma-specific outcomes, it is also possible that prenatal exposure to heightened stress hormone resulted in a more generalized programming of innate immunity and inflammation in offspring. Because HDM preparations contain endotoxin, the enhanced inflammation associated with prenatal stress hormone exposure could be the result of a more robust innate TLR4-mediated inflammatory response in the lung. Additional studies examining overall innate immune function in offspring from control and CORT-treated dams will address these ongoing questions about how prenatal stress hormone is effecting fetal immune development and postnatal immune functions.

## Data Availability Statement

The raw data supporting the conclusions of this article will be made available by the authors, without undue reservation, to any qualified researcher.

## Ethics Statement

The animal study was reviewed and approved by Michigan State University Institutional Animal Care and Use Committee.

## Author Contributions

AS performed and designed experiments, performed analyses, and aided in manuscript writing. EP performed and analyzed experiments, and aided in manuscript preparation. DM, RS, and EF performed experiments, aided in analysis, and manuscript preparation. DJ-H and JH aided in development of allergy model, performed histological analysis, and aided in manuscript preparation. KR developed project idea, oversaw design and performance of experiments, performed data and statistical analyses, and oversaw manuscript preparation.

### Conflict of Interest

The authors declare that the research was conducted in the absence of any commercial or financial relationships that could be construed as a potential conflict of interest.
